# Local NMDA Receptor Blockade Attenuates Chronic Tinnitus and Associated Brain Activity in an Animal Model

**DOI:** 10.1371/journal.pone.0077674

**Published:** 2013-10-21

**Authors:** Thomas J. Brozoski, Kurt W. Wisner, Boris Odintsov, Carol A. Bauer

**Affiliations:** 1 Division of Otolaryngology, Southern Illinois University School of Medicine, Springfield, Illinois, United States of America; 2 Biomedical Imaging Center, Beckman Institute for Advanced Science and Technology, University of Illinois, Urbana Champaign, Urbana, Illinois, United States of America; University of Regensburg, Germany

## Abstract

Chronic tinnitus has no broadly effective treatment. Identification of specific markers for tinnitus should facilitate the development of effective therapeutics. Recently it was shown that glutamatergic blockade in the cerebellar paraflocculus, using an antagonist cocktail was successful in reducing chronic tinnitus. The present experiment examined the effect of selective N-methyl d-aspartate (NMDA) receptor blockade on tinnitus and associated spontaneous brain activity in a rat model. The NMDA antagonist, D(−)-2-amino-5-phosphonopentanoic acid (D-AP5) (0.5 mM), was continuously infused for 2 weeks directly to the ipsilateral paraflocculus of rats with tinnitus induced months prior by unilateral noise exposure. Treated rats were compared to untreated normal controls without tinnitus, and to untreated positive controls with tinnitus. D-AP5 significantly decreased tinnitus within three days of beginning treatment, and continued to significantly reduce tinnitus throughout the course of treatment and for 23 days thereafter, at which time testing was halted. At the conclusion of psychophysical testing, neural activity was assessed using manganese enhanced magnetic resonance imaging (MEMRI). In agreement with previous research, untreated animals with chronic tinnitus showed significantly elevated bilateral activity in their paraflocculus and brainstem cochlear nuclei, but not in mid or forebrain structures. In contrast, D-AP5-treated-tinnitus animals showed significantly less bilateral parafloccular and dorsal cochlear nucleus activity, as well as significantly less contralateral ventral cochlear nucleus activity. It was concluded that NMDA-mediated glutamatergic transmission in the paraflocculus appears to be a necessary component of chronic noise-induced tinnitus in a rat model. Additionally, it was confirmed that in this model, elevated spontaneous activity in the cerebellar paraflocculus and auditory brainstem is associated with tinnitus.

## Introduction

Chronic tinnitus, or “ringing in the ears,” is experienced by 10 to 40 percent of the human population, depending on survey methodology and demographics. For 3 to 5 percent of those affected, it significantly and negatively impacts quality of life [1]. Although palliative treatments exist, there is no broadly effective treatment that reliably reduces or eliminates the sensation. This is surprising, given that tinnitus is typically a low-level sensation with simple qualitative features. The failure to adequately treat tinnitus may be attributed to an incomplete understanding of the disorder's pathophysiology.

Although not everyone with tinnitus has clinically-defined hearing loss, most do, and many have historical and/or audiological evidence of damaging sound exposure [2]. High-level sound exposure has many long-term consequences for auditory processing. Among them are altered stimulus-driven activity in peripheral afferents, including frequency-specific decreases as well as increases [3], and complex alterations of neural activity along the central auditory pathway, generally characterized by compensatory increases [4]. One widely-considered hypothesis is that the central auditory pathology in tinnitus is driven by a down regulation of inhibition [5]. In support of this hypothesis, both direct [6] and indirect evidence [7], [8], [9] indicates that gamma amino-butyric acid (GABA) mediated inhibition is decreased in the long term following exposure to damaging acoustic events. However, throughout the nervous system, and particularly in the auditory system [10], [11] excitatory and inhibitory functions are coupled in dynamic balance. Given this coupling, increased excitation would be expected to accompany inhibitory downregulation. In support of an excitatory tinnitus component, peripheral [12] and central glutamatergic upregulation [6], [13] have been demonstrated in experimental animals with tinnitus or following auditory insult. At present it is unclear whether compensatory central alterations leading to tinnitus are driven primarily by a down regulation of inhibition, an up regulation of excitation, or whether both occur in tandem.

Traditionally the cerebellum is described as a control system that uses multi-modal sensory information to fine tune motor action [14]. Contemporary research has expanded that view to include perceptual gating and memory functions [15]. In the rodent brain, the cerebellar flocculus (FL) and paraflocculus (PFL) are in close proximity to the dorsal cochlear nucleus (DCN). The FL, PFL and DCN have similar neural circuitry [16] and may have similar functions in adaptive auditory signal processing. Using classical anatomical methods, Rasmussen showed that first- and second-order auditory afferents enter the PFL [17]. Morest and colleagues were the first to demonstrate degeneration of auditory afferents within the PFL and cerebellar stalk after cochlear damage[18]. Notably, degenerating auditory afferents terminated in the transition zone of the cerebellar FL, an area containing high densities of doublecortin positive unipolar brush cells (UBCs). More recently, using functional imaging methods, PFL activity was shown to be elevated in rats with psychophysical evidence of tinnitus, but not in rats with normal hearing exposed to an external tinnitus-like sound [19]. This suggests that PFL circuits, perhaps in conjunction with those of the DCN, provide compensatory mechanisms for peripheral auditory damage. Overcompensation or aberrant compensation may be responsible for tinnitus.

The interplay of GABAergic and glutamatergic processes in the cerebellum is well documented. The role of UBCs can be placed within this context. UBCs are local-circuit glutamatergic interneurons, found in considerable density in the granule cell layer of the rat DCN, FL and PFL. UBCs receive glutamatergic excitatory inputs from extrinsic mossy fibers as well as from intrinsic UBCs. They have been hypothesized to comprise local networks of tunable feed-forward amplifiers [20], [21]. In the DCN, they might also participate in feed-back amplification from higher auditory centers. UBC circuits are therefore situated to mediate compensatory signal gain after afferent damage. Recently it was shown that UBCs in the PFL and DCN of adult rats express doublecortin, a marker of migrating and immature neurons [22]. Enhanced doublecortin immunoreactivity has been shown in the DCN, FL and PFL of rats with tinnitus [23]. In the same experiment it was shown that direct PFL infusion of combined glutamatergic antagonists attenuated tinnitus (D-AP5, D(−)-2-Amino-5-phosphonopentanoic acid; and CNQX, 6-Cyano-7-nitroquinoxaline-2,3-dione disodium salt hydrate), while infusion of combined glutamatergic agonists produced tinnitus-like symptoms in normal control animals (s-AMPA, (S)-α-Amino-3-hydroxy-5-methylisoxazole-4-propionic acid; and NMDA, N-Methyl-D-aspartic acid). These results are consistent with an excitatory glutamatergic component of chronic tinnitus, mediated by UBCs in the PFL.

The present experiment examined the effect of PFL infusion of the selective glutamatergic N-methyl d-aspartate (NMDA) receptor antagonist, D(−)-2-amino-5-phosphonopentanoic acid (D-AP5), on chronic acoustic-trauma-induced tinnitus in rats. If tinnitus emerges as a result of neuroplastic overcompensation, then NMDA receptors might be an important substrate via their established role in neuroplasticity. The objective of the present research was to examine the contribution of NMDA receptors in the PFL to chronic tinnitus, and to further examine tinnitus-associated brain activity in the auditory pathway and areas of high UBC density.

## Materials and Methods

### Ethics statement

This study was carried out in strict accordance with the recommendations in the Guide for the Care and Use of Laboratory Animals of the National Institutes of Health. The experimental protocol was approved by the Laboratory Animal Care and Use Committee of Southern Illinois University School of Medicine.

### Subjects and group composition

16 male Long-Evans rats (Harlan, Indianapolis, IN, USA) 60 days old at the outset of the study, were individually housed and maintained at 25°C with a 12/12 h reversed light/dark schedule. The 16 were selected from 42 rats, of identical age, sex, and history, engaged in a larger tinnitus experiment. The selection was made when the animals were approximately 8 months old and 3 months after they had been trained and tested for tinnitus. Selection was determined by their experimental exposure and psychophysical performance: 11 of 16 were exposed to tinnitus-inducing sound (described below) and displayed significant evidence of tinnitus; 5 of 16 were not exposed to tinnitus-inducing sound and had no evidence of tinnitus. Of the 11 rats with tinnitus, 6 were treated with D-AP5 and 5 were untreated. An unexposed D-AP5 treated group was not included because unilateral PFL surgical ablation, a more radical intervention than NMDA blockade, was shown in previous research not to affect performance of unexposed rats on the psychophysical task used in the present experiment [24].

### Tinnitus Induction

At 90 days of age, 11 animals were anesthetized with a 1.7 percent isoflurane /O_2_ mixture, placed in a modified head holder with a speaker driver (FT17H, Fostex, Tokyo, Japan) attached to 2 cm length of flexible tubing positioned at the entrance of the right ear canal. They were unilaterally exposed once for 1 hr to band-limited noise, with a peak level of 120 dB (SPL) centered at 16 kHz and falling to ambient levels at 8 kHz and 24 kHz. ABR thresholds were obtained before and after exposure (described below). The exposure preceded behavioral training. The contralateral canal was obstructed by the canal-fitted plastic tube from the contralateral speaker. Details of the trauma exposure have been previously published [25], [26]. The exposure temporarily elevated auditory brainstem evoked response (ABR) thresholds, 30 – 50 dB in the exposed ear, while leaving the contralateral ear unaffected in all animals. The 11 exposed rats in this experiment all developed significant tinnitus, and were specifically selected from a larger pool of noise-exposed animals. This level of sound exposure has been shown to leave both inner and outer hair cells largely intact, although ipsilateral loss of large-diameter spiral ganglion dendrites across a broad frequency range is evident [27].

### Hearing Thresholds

ABR thresholds were obtained immediately before and after exposure, and at the conclusion of psychophysical testing, 8 months after the start of the experiment. Unexposed animals were similarly tested without an immediate post-exposure measurement. ABR measurements were obtained using a customized TDT System 3 (Tucker Davis Technologies, Alachua, FL, USA). Acoustic stimuli were presented directly to the entrance of the ear canal. A stainless steel needle electrode was placed subcutaneously at the vertex and a reference electrode at the occiput. ABR thresholds were obtained for 5 msec duration tone bursts presented at a rate of 20/sec. Tone bursts were gated using an cosine envelope (2.5 msec rise/decay, 0 msec plateau) and were presented in decreasing intensity series (10 dB decrements, starting at 95 db, SPL). Threshold was determined by the lowest intensity that produced visually and statistically distinct evoked waveforms within a 12 msec peri-stimulus window. Evoked potentials (X 200,000) were averaged over 512 sweeps, digitized with 4 µsec resolution, and imported to custom spreadsheets (Excel, Microsoft, Redmond, WA, USA) for statistical analysis. Spreadsheet algorithms enabled evoked responses to be distinguished from baseline, determined by RMS amplitude.

### Calibration

Sound levels were calibrated using a Bruel & Kjaer Pulse sound measurement system (Pulse 13.1 software), equipped with a 3560C high-frequency module. Exposure levels and ABR levels were determined using a 4138 pressure-field microphone (Brüel & Kjaer, Norcross, GA, USA) coupled to the output transducers using rubber tubing with internal dimensions approximately matching that of an adult rat external auditory canal. Linear sound level determination was possible between 5 and 140 dB (SPL re 20 µPa), and from 5 Hz to 104 kHz. Sound levels in the behavioral test chambers were calibrated using a B&K 4191 open-field microphone. All levels reported are unweighted.

### Tinnitus Assessment

Tinnitus was determined using a behavioral procedure demonstrated to be sensitive to tinnitus, described elsewhere [25], [26]. Briefly, an operant conditioned-suppression procedure was used to characterize the animals' perception of tones and silent periods presented in the context of ambient 60 dB (SPL) broad-band noise (BBN). The animals were required to discriminate between the presence and absence of sound when tested with a variety of sounds of different composition, frequency, and level. While the features of tinnitus in rats (and other species) cannot be directly known, by definition tinnitus cannot sound like silence. Animals were tested daily in operant test chambers (Lafayette Instruments, Mod. 80001, Lafayette, IN, USA) equipped with lid-mounted speakers. Speaker-off periods (i.e., silence) acquired significance for the animals because lever pressing during the silent periods led to a foot shock at the end of the period (0.5 mA, 1 sec duration). Foot shock could be avoided by not lever pressing (i.e., suppression). With the exception of silent periods, lever pressing never led to foot shocks. The behavior of interest was lever pressing during randomly inserted test sounds (1 min duration) that substituted for some of the speaker-off presentations (also 1 min duration). A one hour test session contained 10 randomly inserted, non-contiguous presentations of sounds or silence, with the background BBN off. Two of the ten were always silent (i.e., speaker off) periods. The remaining 8 were of a randomly-selected tone or noise, with different levels in each presentation. The decision task for the animals was to discriminate between the test stimulus and speaker off. Lever pressing was quantified using a relative rate measure, the suppression ratio (R). R was determined as a running measure for successive 1-min segments of each session using the formula R = B/(A+B), where A was the number of lever presses in the preceding 1-min segment and B the number of lever presses in the current 1-min segment. R can vary between 0 and 1. A value of 0 is attained when lever pressing in the current minute is 0, a value of 0.5 when lever pressing in the current minute is equal to that of the previous minute, and a value of 1 when lever pressing in the previous minute is zero. R provided a running index of behavior, in 1-min epochs, and enabled a quantitative comparison between subjects as well as unbiased compilation of group data. R is a useful index of perceptual performance since it is sensitive to short-term behavioral effects, such as those produced by sensory events, but it is insensitive to gradual behavioral effects, such as those produced by changes in motivational status, for example, satiation. In the context of the present procedure, it was expected that exposed rats with tinnitus would have lower R-values than unexposed rats, when tested with stimuli that resembled their tinnitus. Further details of the psychophysical procedure are available in an open-access document [26].

### Drug delivery

The drug delivery method was designed to continuously infuse the brain area of interest (AOI) with D-AP5 (Tocris Bioscience, Boston, MA, USA) for the time required to complete psychophysical testing (i.e., 2 weeks). A sterile solution of D-AP5 (0.5 mM, normal saline, pH 7) was loaded into osmotic mini pumps (Alzet Model 2002, Durect Corp., Cupertino, CA, USA). Each pump delivered 0.5 µl per hour for 14 days. Under aseptic conditions, a single pump was placed subcutaneously in the caudal neck region while under isoflurane anesthesia (2% at 0.8 L/min). The pump was anchored to subdermal muscle and connective tissue using a nylon suture. Pump output was delivered via a length of PE-50 catheter tubing, the end of which fit tightly into a 1 mm craniotomy made in the subarcuate fossa, above the dorsal aspect of the right PFL (ipsilateral to the noise-exposed ear). A flare formed at the distal catheter tip made a tight seal within the craniotomy, and the catheter was further sealed to the skull surface using bone wax and thermal polyethelene adhesive (Best Stik, FPC Corp., Wauconda, IL, USA). The catheter tip terminated in subdural space proximal to the surface of the PFL. The skin incision was closed with wound clips and the animals were allowed to recover for 48 hrs before psychophysical testing resumed. Fifteen days after implant, the animals were again anesthetized and their catheters and pumps were removed. At the time of removal, both the position and patency of the catheters was confirmed. In addition, the pumps were inspected to confirm that their drug reservoirs were empty. All six implanted animals met placement/patency criteria. Psychophysical testing for tinnitus resumed after a 48 hr recovery period. As before, all animals were tested in parallel, with testing extending for 23 days after drug pump removal. At the conclusion the animals were scheduled for manganese enhanced magnetic resonance imaging (MEMRI) scans.

A control procedure, using identical pumps and catheters, filled with methylene blue, was used to check diffusion of the infused solution. Macro photographic and micrographic examination confirmed that solution diffusion was limited to the ipsilateral PFL, and did not enter the adjoining brainstem [23].

### MEMRI

Prior to MEMRI acquisition, ABR threshold measurements were repeated. One unexposed control and one exposed non-drug animal expired from anesthetic overdose and were not imaged. MEMRI scans were obtained from 6 exposed D-AP5-treated animals, 4 exposed non-drug-treated animals, and 4 unexposed-untreated animals, 8 weeks after conclusion of D-AP5 treatment. Animals were imaged in batches of 4 to 6 animals, with the batches composed of animals from all treatment groups.

Magnetic resonance images were acquired on a ultra-high resolution vertical bore (89 mm) micro-imaging scanner (Oxford Instruments, Abington, UK) equipped with a Unity/Inova console (Varian, Palo Alto, CA), operating at 14.1 T. The 600 MHz NMR spectrometer was equipped with four identical receiver channels and gradient coils with maximum strength of 95 Gauss/cm. A high image resolution Spin-Echo-Multi-Slice (SEMS) protocol was employed for T1-weighted acquisition. Short repetition time was used to increase T1 contrast. Axial T1-weighted images were acquired slice by slice with no gap between slices using the following parameters:

• repetition time (TR) = 350 ms,

• echo time (TE) = 10 ms,

• number of transients averaged (NT) = 20,

• spectral width (SW) = 71 kHz,

• matrix size = 256×256,

• number of slices = 26,

• slice thickness of 0.3 mm,

• field-of-view (FOV) = 25×25 mm^2^.

In T1-weighted images, Mn2+ contrasted areas appear as hyperintense regions. Receiver gain and radiofrequency transmit power were optimized utilizing VnmrJ software. The average scan time for a full image set was 29 min. Images were processed using custom-written Matlab codes that used a signal-to-noise normalization segment to reduce average contrast between slices and improve image quality. Images from individual animals were captured with parameters held constant. A patented tunable transmit/receive radiofrequency (RF) coil was used in for image acquisition [28]. The RF coil reduced limits imposed by dielectric tissue properties and other losses (e.g., water) and expanded the resonance frequency tuning range up to 30 percent (c.f., 1-3% typical for commercial coils) while maintaining appropriate impedance matching. Coil electrical symmetry was preserved across the entire tuning range, resulting in high Q-factor and RF field homogeneity inside the coil. Unprocessed image-plane resolution was 100 µm, while interpolated image-plane resolution was 25 µm.

Approximately 20 hrs prior to imaging, the animals received 80 mg/kg, SC, of MnCl_2_ dissolved in sterile saline. They were returned to their home cage, with food removed, and placed in a double-wall acoustic isolation booth without sound. The objective was to equate the acoustic environment, and minimize ambient sound stimulation of all animals prior to imaging. Animals in each batch were imaged in random order. Immediately prior to imaging animals were given a lethal anesthetic dose (Euthasol, Virbac, Ft. Worth, TX, USA), decapitated, the mandible removed, and excess muscle tissue dissected away from the skull. The head was placed in a polyethylene holder along with a 1 mm diameter glass capillary filled with CuSO4 (3 mM). The image phantom of the capillary indexed the left hemisphere, making image laterality unambiguous.

**Figure 1:**
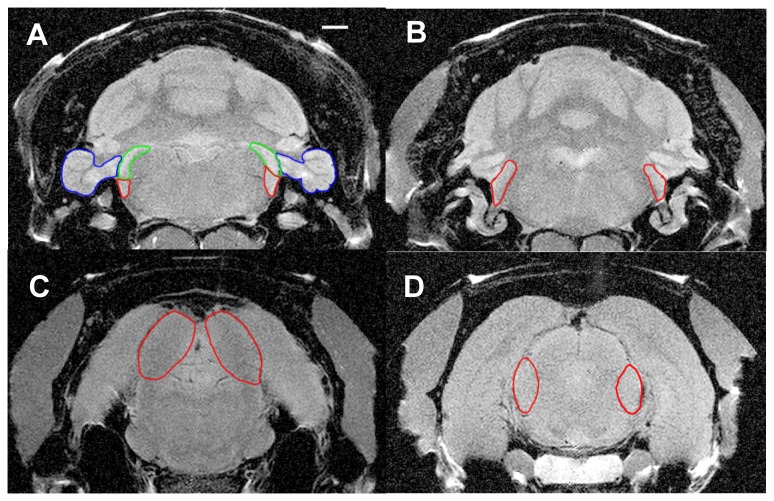
Typical AOI boundaries used in MEMRI quantification. A. Paraflocculus (PFL) blue, dorsal cochlear nucleus (DCN) green, posterior ventral cochlear nucleus, red. The 2 mm scale bar applies to all panels. B. Anterior cochlear nucleus (AVCN). C. Inferior colliculus (IC). D. Medial geniculate body (MGB).

Scans were converted to 32-bit grayscale TIFF images, and imported into Image J (1.44 p, http://imagej.nih.gov/ij) for analysis. AOI were determined using visible landmarks, such as the auditory nerve, in combination with digital overlays from a rat brain atlas [29]. AOI included the PFL, the DCN, the anterior and posterior ventral cochlear nucleus (AVCN and PVCN, respectively), the inferior colliculus (IC) and the medial geniculate body (MGB). Left and right hemispheres were measured separately. Each AOI was outlined by an experimenter, blind to the animals' treatment history, taking care to avoid occasional image anomalies of unknown origin. Typical AOI outlines are shown in Fig. 1. AOI brightness levels within each outline were digitized using the Image J level tool and entered into spreadsheets for analysis. Brightness was also determined for a muscle mass proximal to each AOI, and these values were used as a reference level for each AOI. Using the muscle brightness reference, each AOI level was converted to a ratio score (AOI level / muscle level), thereby eliminating the potential confound of global image brightness variation. AOI neural activity was assumed to be proportional to relative brightness, which in turn depended upon Mn uptake. Total AOI activity level was represented by an average derived from all slices intersecting that AOI. Using these, averages were derived for each treatment group. Treatments were compared using mixed ANOVAs, with group treatment as the independent factor and AOI as the repeated-measures factor.

## Results

### Hearing thresholds

**Figure 2:**
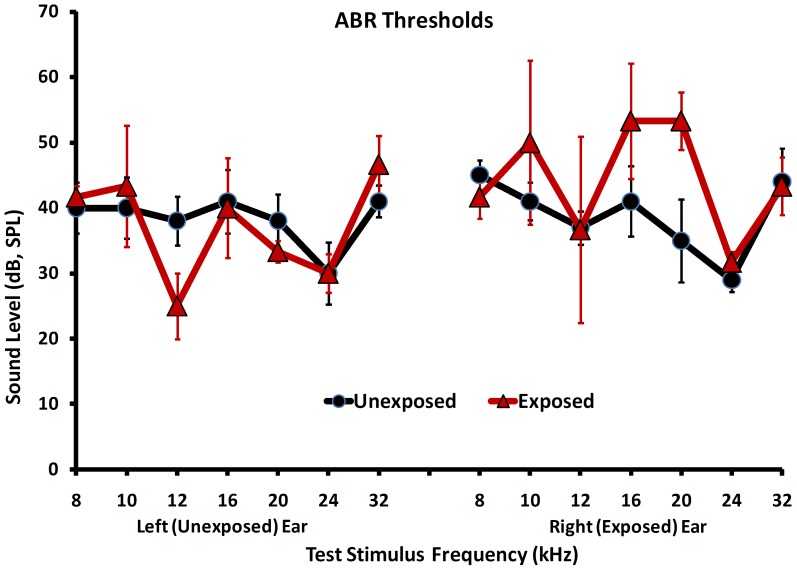
Pre-imaging, immediate post-psychophysics, auditory brainstem response (ABR) hearing thresholds for Exposed (see text) and Unexposed groups. Error bars indicate the standard error of the mean.

At the conclusion of psychophysical testing, ipsilateral ABR thresholds in exposed animals were elevated 12 to 18 dB at 16 and 20 kHz, respectively (Fig. 2). Ipsilateral thresholds at other frequencies, and at all contralateral thresholds, did not differ from those of unexposed controls. Across test frequencies, ABR thresholds for unexposed animals were clustered around 40 dB (SPL) (Fig. 2).

### Psychophysical evidence of tinnitus

**Figure 3:**
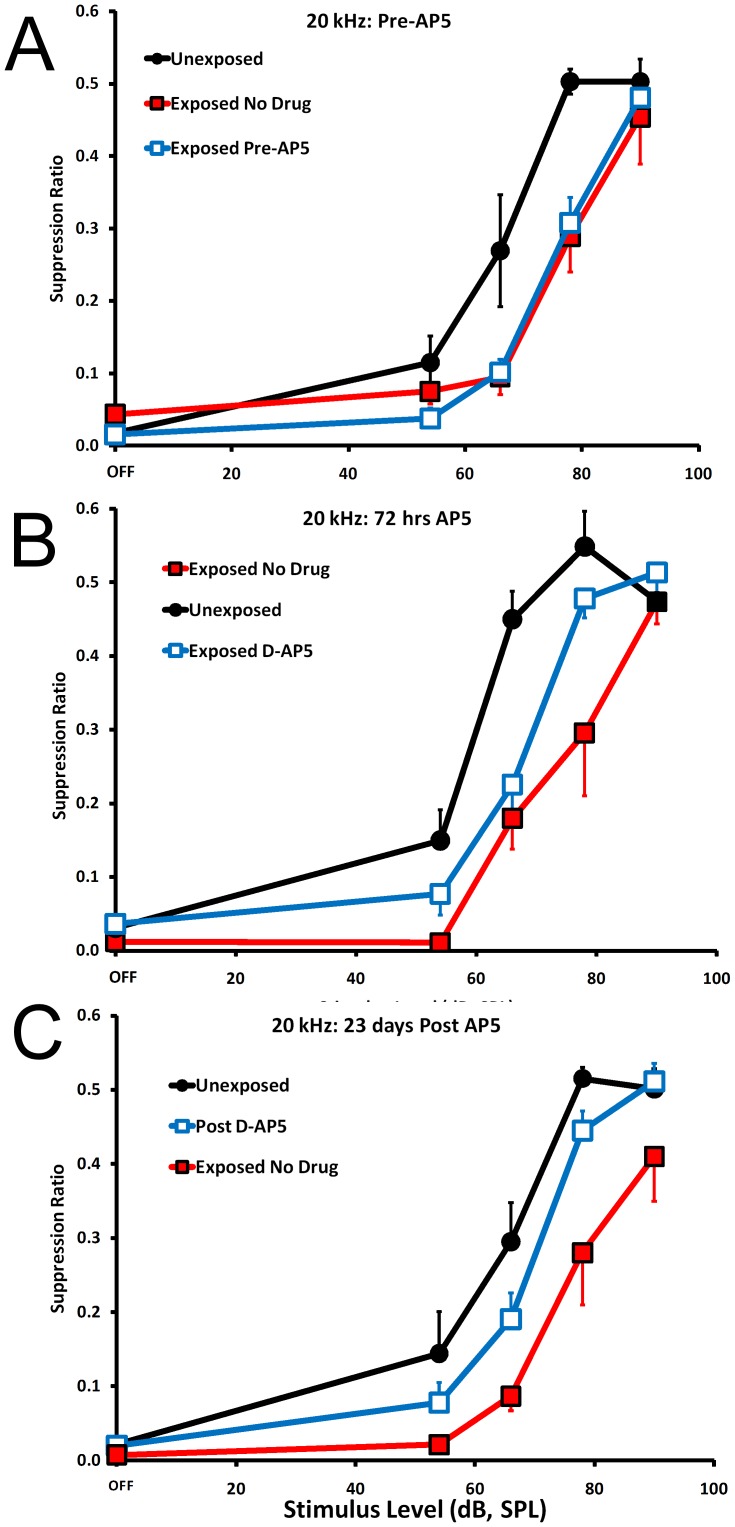
Psychophysical performance of Exposed (square data points) and Unexposed (round data points) groups. Prior to D-AP5 treatment, exposed animals were divided into two equal subgroups, one of which was randomly assigned to receive D-AP5 (open, square data points). A downward shift with respect to Unexposed controls was indicative of tinnitus. **A**. Pre-D-AP5 treatment there was no difference between the two exposed groups (error bars in all panels indicate the standard error of the mean) and both were significantly different than the Unexposed group (statistics summarized in Table 1, section 1). **B**. After 72 hrs of D-AP5 infusion the treated Exposed group significantly diverged from the untreated Exposed group (statistics summarized in Table 1, section 2), indicating decreased tinnitus. **C**. The D-AP5 treatment effect persisted for 23 days after discontinuation of drug infusion (statistics summarized in Table 1, section 3).

Prior to drug treatment, compared to unexposed controls, exposed rats in both groups (drug treated and untreated) showed equivalent and significantly downshifted 20 kHz discrimination functions (Fig. 3A; statistics summarized in Table 1). In this paradigm, when tinnitus is induced before training and testing, a downshift is indicative of tinnitus [25]. Mixed ANOVAs were used to determine significance levels. Treatment groups was the independent factor and stimulus level (e.g., 30, 40, 50 60 dB) was the repeated measure. Each stimulus test condition (e.g., BBN, 20 kHz) and experimental condition (pre-drug, on drug, post drug) was treated as a distinct experiment.

**Table 1:** Anova summary of psychophysical data (20 kHz diagnostic stimulus).

*Source of Variation*	*SS*	*df*	*MS*	*F*	*P-value*
**1. Sound exposure effect: Before D-AP5, each Exposed group differed from Unexposed.**					
a) Pre-D-AP5-Exposed vs Unexposed	0.1598	1	0.1598	26.10	8.34E-06
Error	0.2449	40	0.0061		
b) Untreated Exposed vs Unexposed	0.1424	1	0.1424	14.16	0.000676
Error	0.3216	40	0.0100		
**2. Initial therapeutic effect: D-AP5 (day 3)+Exposed differed from a) Untreated exposed, and from b) Untreated unexposed.**					
a) D-AP5+Exposed vs Untreated Exposed	0.0833	1	0.0833	9.80	0.003248
Error	0.3399	40	0.0084		
b) D-AP5+Exposed vs Untreated Unexposed	0.0813	1	0.0813	11.24	0.001753
Error	0.2893	40	0.0072		
**3. Long-term therapeutic effect: D-AP5 (day 14)+Exposed continue to differ from: a) untreated exposed, but no longer differ from b) unexposed (and untreated) controls (two highest sound test levels).**					
a) D-AP5+Exposed vs Untreated Exposed	0.0507	1	0.0507	7.99	0.010394
Error	0.1270	20	0.0063		
b) D-AP5+Exposed vs Untreated Unexposed	0.0166	1	0.0166	3.04	0.096203
Error	0.1095	20	0.0054		

### D-AP5

Three days after receiving the D-AP5 implant, the treated animals showed decreased tinnitus, but not complete remission (Fig. 3B). That is to say, their diagnostic 20 kHz discrimination function was significantly upshifted from the untreated exposed animals, but still significantly downshifted from unexposed controls (Fig. 3B; Table 1, section 2). After 14 days of continuous D-AP5 infusion, and extending to 23 days after the cessation of infusion, the treatment effect of D-AP5 was still evident (Fig. 3C). Considering the two highest test stimulus levels (78 and 90 dB, SPL), there was no significant difference between the exposed-D-AP5-treated and unexposed control animals (Table 1, section 3). Therefore, a 14 day infusion of D-AP5 (0.5 mM, 2 µL / hr) to the paraflocculus ipsilateral to the exposed ear significantly reduced acoustic-trauma-induced tinnitus. Furthermore, the treatment effect persisted for more than three weeks after discontinuation of the drug.

### MEMRI

**Figure 4:**
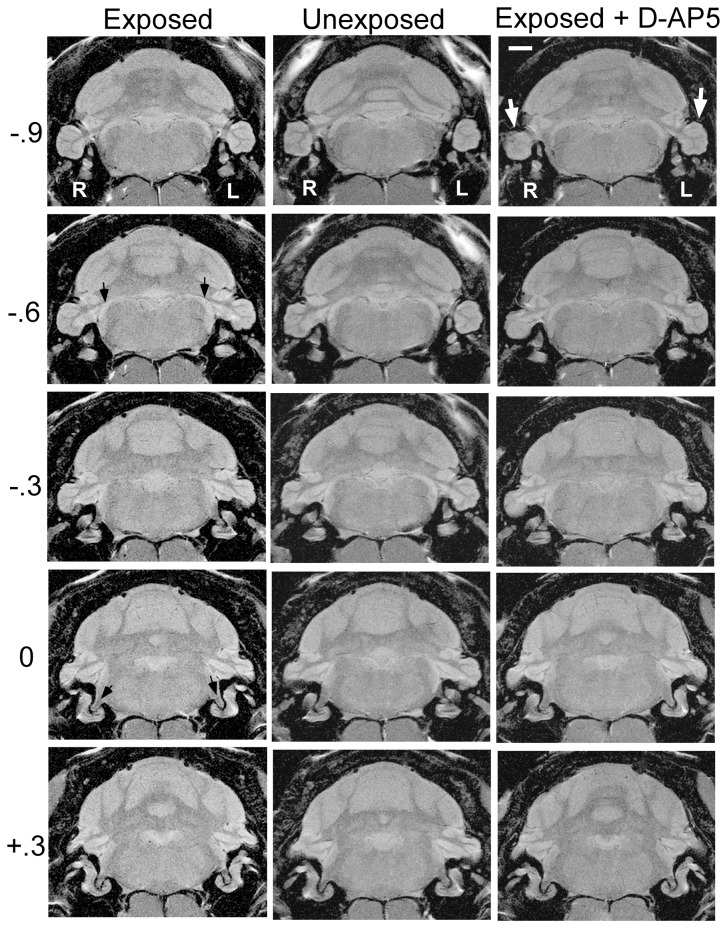
Representative MEMRI scans of an exposed (tinnitus) untreated rat (left column), an unexposed (no-tinnitus) untreated rat (center column), and an exposed treated rat (right column). Panel rows are indexed with respect to the auditory nerve (dark arrows in row 0). The row labels indicate distance in mm rostral (positive values) or caudal (negative values) to the auditory nerve. The dorsal cochlear nucleus (dark arrows) appears in row −0.6 and the paraflocculus (white arrows) in row −0.9. The scale bar (top right panel) is 2 mm, R and L indicate the right and left hemispheres, respectively.

**Figure 5:**
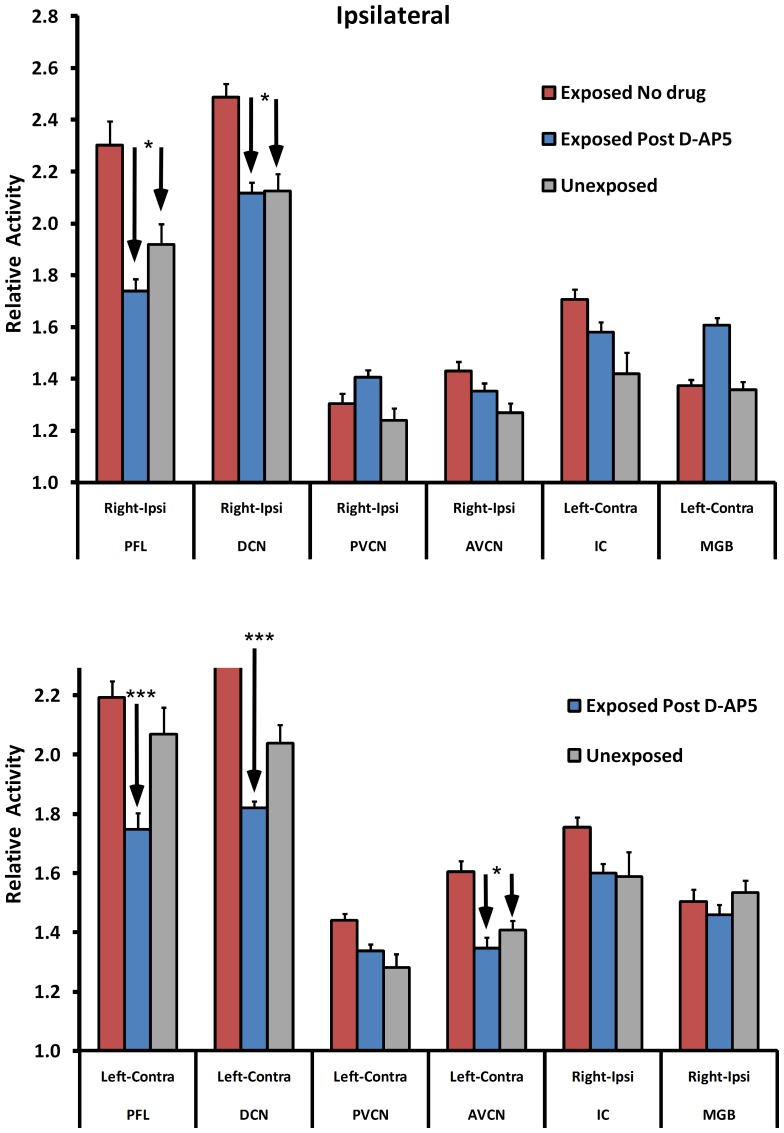
Post D-AP5 spontaneous neural activity indicated by manganese enhanced magnetic resonance imaging (MEMRI). Depicted are normalized data (AOI / adjacent muscle area). Elevation was evident in the ipsilateral paraflocculus (PFL) and dorsal cochlear nucleus (DCN) of Exposed untreated, but not in Exposed D-AP5 treated animals, in comparison to Unexposed controls (top panel). Contralateral elevation (bottom panel) was evident in the PFL, DCN, and the anterior ventral cochlear nuclei (AVCN). Posterior ventral cochlear nucleus (PVCN), inferior colliculus (IC), medial geniculate body (MGB). Error bars indicate mean deviation (*** p<0.0001; * p<0.05); statistical summary in Table 2.

Five weeks after the conclusion of psychophysical testing, and eight weeks after the conclusion of D-AP5 treatment, neural activity was determined in auditory brainstem and midbrain AOI (see Methods) using MEMRI. Representative images are shown in Fig. 4 for an exposed-untreated animal (Fig. 4 left column), an unexposed (and untreated) animal (Fig. 4 center column), and an exposed-D-AP5-treated animal (Fig. 4 right column). The sections, 0.3 mm thick, are indexed with respect to the auditory nerve (Fig 4., dark arrows, row 0, left column), and extend from the cochlear nucleus (Fig. 4, dark arrows, row -.6, left column) through the PFL (Fig. 4, light arrows, row -.9, right column). Average group results are summarized in Fig. 5 (top panel) for the ipsilateral pathway, and in Fig. 5 (bottom panel) for the contralateral pathway. Three significant effects were obtained (statistics summarized in Table 2): (a) Neural activity was elevated in the exposed untreated DCN and exposed untreated PFL, in comparison to unexposed controls (Table 2, section I). This was evident both ipsilateral (Fig. 5, top panel) and contralateral (Fig. 5, bottom panel) to the exposed ear. (b) Bilateral neural activity in the DCN and PFL of D-AP5 treated exposed animals, was equivalent to that of unexposed (non-tinnitus) controls (Table 2, section 2A), and significantly lower than that of exposed untreated (tinnitus) animals (Table 2, section 2B). (c) Contralateral anterior ventral cochlear nucleus activity was elevated in exposed untreated rats, while in exposed D-AP5 treated rats it was comparable to unexposed. D-AP5 was therefore effective not only in decreasing the psychophysical evidence of tinnitus, but also effective in reducing PFL and DCN activity to normal control levels for more than two months post treatment. Global muscle levels were not significantly different between treatment groups (exposed D-AP5: 0.185±0.056; exposed no drug: 0.206±0.029; unexposed: 0.1809±0.033; p = 0.27 to 0.90). These results demonstrate that a two-week unilateral parafloccular infusion of D-AP5 had a long lasting treatment effect on chronically-established tinnitus and that this effect was associated with a bilateral reduction of PFL and auditory brainstem spontaneous activity.

**Table 2:** Anova summary of MEMRI data.

*Source of Variation*	*SS*	*df*	*MS*	*F*	*P-value*
**1. Sound exposure effect: Ipsilateral PFL & DCN Activity** **a) Untreated Exposed (no drug) differed from Unexposed;** **b) PFL & DCN were equivalent.**					
Untreated Exposed vs Unexposed	0.4313	1	0.4303	5.14	0.0427
PFL vs DCN	0.1841	1	0.1841	2.19	0.1645
Interaction	0.0038	1	0.0038	0.05	0.8352
Error	1.0079	12	0.0840		
**2. Drug treatment effect: Ipsilateral PFL & DCN Activity** **A. D-AP5+Exposed do not differ from Unexposed;** **B.D-AP5+Exposed differ significantly from Untreated Exposed.**					
A. D-AP5+Exposed vs Unexposed	0.1150	1	0.1150	0.56	0.4644
PFL vs DCN	0.5843	1	0.5843	2.82	0.1083
Interaction	0.0267	1	0.1291	0.72	0.4351
Error	4.1352	20	0.2068		
B. D-AP5+Exposed vs Untreated Exposed	1.3074	1	1.3074	6.26	0.0211
PFL vs DCN	0.4746	1	0.4746	2.27	0.1472
Interaction	0.0571	1	0.0571	0.27	0.6068
Error	4.1738	20	0.2087		

## Discussion

Chronic tinnitus commonly emerges as a consequence of damage to the peripheral auditory system. Homeostatic mechanisms appear to compensate for lost afferent input, and these mechanisms may over compensate [4], resulting in the sensation of sound when no acoustic signal is present. The involvement of NMDA receptors in chronic tinnitus has been considered both in the context of basic research and clinical treatment, with mixed results. Guitton et al., [12] identified NMDA-receptor mediation of tinnitus in the cochleas of salicylate treated rats. An NMDA antagonist (MK-801) applied to the round window of the cochlea, was effective in decreasing tinnitus in their model. In a follow-up experiment Guitton and Dudai [30] used a different animal model and different NMDA antagonist, as well as an AMPA antagonist. An important difference in their second model was the inclusion of acoustic exposure-induced tinnitus. Both NMDA and AMPA peripheral blockade (antagonists directly applied to the cochlear round window) were effective in preventing tinnitus, when applied before exposure, but only NMDA block displayed a therapeutic rescue effect when applied after exposure. In total, these results suggest that cochlear NMDA receptor activity may be more relevant to emergence of tinnitus pathology than AMPA-mediated glutamatergic transmission. Lobarinas et al., using a salicylate/quinine rat model, reported that systemic memantine (1.5 or 3 mg/kg/day), a non-competitive NMDA antagonist, was not effective in reducing the salicylate-induced tinnitus [31]. Salicylate models of tinnitus, however, are acute, and the high doses of salicylate or quinine used, raise questions about mechanisms relevant to those underpinning chronic acoustic-exposure-induced tinnitus. In a more recent study, systemic administration of memantine (5 mg/kg), significantly reduced tinnitus in a rat model of acoustic-trauma-induced tinnitus [32]. The Zheng et al. tinnitus model was similar to that used in the present experiment. They also reported that memantine's therapeutic effect was reversible, disappearing after a washout period [32]. Although memantine studies are informative and of potential clinical relevance, interpretation must be done cautiously because memantine is also active at serotonergic, cholinergic and dopaminergic receptors.

Shulman suggested that glutamatergic antagonists could have a clinical role in tinnitus protection [33]. Blocking glutamatergic transmission would be expected to attenuate the response to acoustic stimuli because auditory first-order afferents are glutamatergic. As a protective strategy, reducing the impact of acoustic overexposure via reduced peripheral sensitivity, is plausible. This type of protection, however raises a functional question, i.e., would protective glutamatergic antagonism be any different than wearing ear protective hardware?

Chronic tinnitus, however, once established, is not dependent upon first-order afferent activity. Tinnitus occurs in the clinical setting of complete auditory nerve transection, illustrating that auditory input is not critical for persisting tinnitus. In the presence of an intact auditory nerve, tinnitus may not even depend upon activity in the DCN [26]. Glutamatergic therapeutic rescue, if it exists, would likely involve neural processing downstream of first-order afferents. The clinical record of glutamatergic antagonists applied to tinnitus rescue treatment is mixed. Oral flupirtine (200 mg/day), a functional NMDA antagonist, was reported to be ineffective in a prospective control open-label clinical trial [34]. Acamprosate, a mixed NMDA antagonist and GABA-A agonist, is widely used to treat alcohol dependence. Two published clinical studies report that acamprosate significantly reduced tinnitus levels. Azevedo and Figueiredo [35], in a placebo-controlled trial, noted that acamprosate (333 mg/day), over 90 days, reduced tinnitus by approximately 4 points on a 10 point rating scale. However this study did not employ a standardized tinnitus evaluation instrument nor did it obtain objective measures of tinnitus loudness. Some of these shortcomings were addressed by Sharma et al., in a study using a blinded double cross-over design and a higher acamprosate dose (333 mg×3/ day) [36]. They reported a significant decrease in subjective tinnitus loudness rating, as indicated by a visual analog scale, and a decrease in objective tinnitus loudness, as indicated by an unspecified loudness matching procedure. They also reported that the improvement in subjective and objective tinnitus symptoms persisted for at least 45 days after discontinuation of drug.

A clinical study using a treatment strategy derived from animal research, applied an experimental NMDA antagonist, AM-101, directly to the cochlear round window of young-adult volunteers with tinnitus [37]. Using a standardized assessment instrument, a visual analog scale of subjective loudness, and minimum masking level objective loudness estimates, they reported a trending improvement on all three scales following a single round-window application. A caution is that those enrolled had short-term tinnitus of less than 3 months duration, and as such they would be expected to have an inflated rate of spontaneous remission. In conclusion, both animal model experiments and clinical tests of NMDA antagonists in treating tinnitus have yielded mixed results.

The DCN and PFL share a similar neural circuitry [16], [38]. Extrinsic excitatory inputs to the DCN are glutamatergic and include primary auditory afferents and mossy fibers that terminate on a variety of cells in the DCN, including fusiform cells, the primary output neurons of the DCN, as well as UBCs and granule cells [16], [21]. Similarly, extrinsic inputs to the cerebellum are glutamatergic and terminate on a variety of cells, including Purkinje cells, the primary output neurons of the cerebellum, as well as UBCs and granule cells [14], [39]. In addition to extrinsic glutamatergic inputs, both the DCN and cerebellum have intrinsic glutamatergic interneurons, the most common of which are granule cells [14] and in regions of the cerebellum such as the FL and PFL, UBCs [22]. Glutamatergic synapses in both the DCN and cerebellum commonly display plasticity (long-term potentiation and depression) and have both AMPA and NMDA receptors. UBCs are uniquely configured to function as fan-out feed forward amplifiers [21] that are modulated by convergent glycinergic and GABAergic inputs [40]. Globally, the cerebellum and DCN appear to exert adaptive control over afferent signals. Adaptive control involves error correction and signal gain adjustment, depending upon multimodal inputs and feedback information. In this context, hearing loss may be characterized as a decrease in afferent information that central systems attempt to correct by increasing gain [4]. The sensation of tinnitus may emerge from these corrective mechanisms. It is plausible that UBCs, as tunable feed-forward amplifiers, participate in the compensatory circuits responsible for tinnitus. Convergent excitatory and inhibitory influences on UBCs could modulate circuit output. Glutamatergic antagonists, such as D-AP5, would be expected to decrease circuit output, as would GABAergic agonists. Previous research, using the present animal model, showed that the GABA agonist vigabatrin, when systemically administered, dramatically reduced tinnitus [8]. More recently, using the same animal model, combined glutamatergic AMPA and NMDA antagonists were shown to reduce tinnitus when micro-quantities were administered directly to the PFL, a region of high UBC density [23].

In the present experiment spontaneous neural activity was quantified using MEMRI. Magnetic resonance imaging (MRI) derives structural information from regional differences in proton density, flow and biochemical structure, which affect local signal levels. In MRI images these variations are seen as either negative (dark) or positive (bright) areas. The present experiment used a 14.1 T magnetic field strength for image acquisition. High magnetic field strength improves nuclear magnetic resonance signal-to-noise ratio and allows the use of smaller voxels, thus benefiting both sensitivity and spatial resolution. Image resolution is further improved through the use of contrast agents. Using Mn2+ as a contrast agent in the present experiment provided a dual benefit: Mn2+ is paramagnetic and reduces longitudinal (spin-lattice) T1 relaxation of nearby water molecules thus improving T1-weighted image contrast. In addition, Mn2+ accumulates in active neurons through voltage-gated calcium channels, primarily at synapses, and therefore serves as a direct functional activity indicator. MEMRI has been used successfully in animal models to document increased brain activity levels in both chronic [19] and acute [41] tinnitus induced by acoustic exposure. In chronic tinnitus the PFL showed the highest relative increase, followed by the IC and VCNp [19]. In acute tinnitus the highest increase was only found in the dorsal cortex of the IC [41]. When acute tinnitus was induced using a high dose of sodium salicylate, elevated activity was also found in the DCN and across all lamina of the IC [41]. In combination these results suggest that the patterns of spontaneous brain activity alterations accompanying tinnitus differ between chronic and acute states, and drug induced versus acoustic-exposure induced tinnitus.

In the present experiment unilateral D-AP5 infused into the PFL not only decreased bilateral spontaneous neural activity, as indicated by MEMRI, in the PFL, but also in the DCN and to a lesser extent in the AVCN (contralateral). It seems unlikely that D-AP5 diffusion into the brainstem could account for the DCN and AVCN decrease, particularly at contralateral sites. This suggests that the cochlear nucleus effects were indirectly derived from NMDA blockade in the PFL. An indirect effect on the DCN is consistent with the hypothesis that the PFL serves as an obligatory generator site for chronic tinnitus. Direct efferent connections between the PFL and the cochlear nuclei have not been documented. However indirect connections are quite plausible. Massive cerebellar efferents to the vestibular brainstem are well documented [15], as well as reciprocal connections to the external nucleus of the IC [42]. The DCN receives substantial input from both the vestibular brainstem [43] and the IC [44]. If the DCN ipsilateral to the treated PFL experienced a loss of excitation, either directly or indirectly from the PFL, that influence could be communicated to the contralateral cochlear nuclei via well-documented contralateral IC connections [42] as well as DCN connections [45] that include glutamatergic contralaterals [46].

The effect of unilateral D-AP5 infusion into the PFL was not examined in unexposed control rats without tinnitus. Previous studies using an identical method of infusion showed that an indicator dye with a diffusion coefficient similar to that of D-AP5 did not spread to areas beyond the parafloccular stalk [47]. It also has been shown that complete unilateral PFL surgical ablation does not affect the psychophysical performance of unexposed control rats, when using the present method to indicate tinnitus [24]. It therefore seems unlikely that D-AP5 action external to the ipsilateral PFL had a significant impact on either tinnitus or on intact auditory performance in the context of the present study.

## Conclusion

Chronic tinnitus appears to depend upon durable plastic changes in central processing. Given the well-established role of NMDA receptors in long-term neuroplasticity, their participation in the plasticity underpinning tinnitus is likely. In the present experiment, the specific NMDA antagonist, D-AP5, delivered directly to the PFL, significantly decreased psychophysically characterized tinnitus. This effect was evident more than three weeks after discontinuation of the drug, suggesting that the compensatory processes leading to tinnitus may have been reset to a pre-exposure state. MEMRI data from D-AP5 treated-and-exposed animals showed PFL and cochlear nucleus activity bilaterally decreased to levels of unexposed animals without tinnitus. Tract-tracing studies have established the presence of both ipsilateral and contralateral fiber projections to the cerebellum [48], in agreement with the present finding of bilateral neural modulation via unilateral drug infusion. The bilateral decrease, and its presence 8 weeks after discontinuation of D-AP5 treatment, support the hypothesis of a system reset to pre-exposure levels. These results suggest that a therapy addressing glutamaterigc circuits, and a specific cell type, may be able to correct the dysfunctional sensation of tinnitus.
